# Identification and characterization of miRNAs and targets in flax (*Linum usitatissimum*) under saline, alkaline, and saline-alkaline stresses

**DOI:** 10.1186/s12870-016-0808-2

**Published:** 2016-05-27

**Authors:** Ying Yu, Guangwen Wu, Hongmei Yuan, Lili Cheng, Dongsheng Zhao, Wengong Huang, Shuquan Zhang, Liguo Zhang, Hongyu Chen, Jian Zhang, Fengzhi Guan

**Affiliations:** Heilongjiang Academy of Agricultural Sciences Postdoctoral Programme, Harbin, 150086 People’s Republic of China; Institute of Industrial Crops, Heilongjiang Academy of Agricultural Sciences, Harbin, 150086 People’s Republic of China; Division of Insect-borne Parastitic Disease Control and Prevention, Harbin Center for Disease Control and Prevention, Harbin, 150056 People’s Republic of China; Alberta Innovates Technology Futures, Vegreville, Alberta T9C 1 T4 Canada

**Keywords:** MicroRNAs, Saline-alkaline stress, Deep sequencing, Degradome, Flax

## Abstract

**Background:**

MicroRNAs (miRNAs) play a critical role in responses to biotic and abiotic stress and have been characterized in a large number of plant species. Although flax (*Linum usitatissimum* L.) is one of the most important fiber and oil crops worldwide, no reports have been published describing flax miRNAs (Lus-miRNAs) induced in response to saline, alkaline, and saline-alkaline stresses.

**Results:**

In this work, combined small RNA and degradome deep sequencing was used to analyze flax libraries constructed after alkaline-salt stress (AS2), neutral salt stress (NSS), alkaline stress (AS), and the non-stressed control (CK). From the CK, AS, AS2, and NSS libraries, a total of 118, 119, 122, and 120 known Lus-miRNAs and 233, 213, 211, and 212 novel Lus-miRNAs were isolated, respectively. After assessment of differential expression profiles, 17 known Lus-miRNAs and 36 novel Lus-miRNAs were selected and used to predict putative target genes. Gene ontology term enrichment analysis revealed target genes that were involved in responses to stimuli, including signaling and catalytic activity. Eight Lus-miRNAs were selected for analysis using qRT-PCR to confirm the accuracy and reliability of the miRNA-seq results. The qRT-PCR results showed that changes in stress-induced expression profiles of these miRNAs mirrored expression trends observed using miRNA-seq. Degradome sequencing and transcriptome profiling showed that expression of 29 miRNA-target pairs displayed inverse expression patterns under saline, alkaline, and saline-alkaline stresses. From the target prediction analysis, the miR398a-targeted gene codes for a copper/zinc superoxide dismutase, and the miR530 has been shown to explicitly target WRKY family transcription factors, which suggesting that these two micRNAs and their targets may significant involve in the saline, alkaline, and saline-alkaline stress response in flax.

**Conclusions:**

Identification and characterization of flax miRNAs, their target genes, functional annotations, and gene expression patterns are reported in this work. These findings will enhance our understanding of flax miRNA regulatory mechanisms under saline, alkaline, and saline-alkaline stresses and provide a foundation for future elucidation of the specific functions of these miRNAs.

**Electronic supplementary material:**

The online version of this article (doi:10.1186/s12870-016-0808-2) contains supplementary material, which is available to authorized users.

## Background

Salt stress is one of the major environmental stresses that limit worldwide agricultural crop yields and will continue to be of concern in future years. In responses to salt stress, such as ionic and osmotic stress, crops have evolved multiple molecular networks to regulate homeostasis and maintain their growth and development. Exposure to salt stress triggers cascades of signal transduction pathways, which induces changes in gene expression profiles [1]. Alkaline-salt stress is generally associated with NaHCO_3_ or Na_2_CO_3_ presence and crops growing in alkaline soils suffer from both high pH stress and CO_3_^2-^/HCO_3_^-^ stress [2]. Therefore, understanding of the saline-alkaline stress response may help to improve crop tolerance to salt stress. However, the mechanisms of plant alkaline-salt tolerance are poorly understood.

MicroRNAs (miRNAs) are endogenous 19–24 nt stretches of noncoding single-stranded RNA that negatively regulate gene expression by inhibiting gene translation or by promoting cleavage of target mRNAs post-transcriptionally [3]. The miRNAs were first discovered in *Caenorhabditise legans* in 1993 [4] and the first plant miRNAs were identified in *Arabidopsis* in 2002 [5]. Recently, several researchers have shown that miRNAs play important roles in plant responses to various abiotic stresses, including low temperature [6], drought [7], high salinity [8], oxidative [9], hypoxic [10], UV-B radiation [11], and metals stress [12]. Additionally, several studies have shown that many differentially expressed miRNAs and their target mRNAs are involved in adaptation to salt stress environments [13]. MiR393 was strongly up-regulated when *Arabidopsis* was treated with 300 mM NaCl, while miR398 was down-regulated under salt stress [14, 15]. In rice, miR169g was shown to be up-regulated during high-salinity stress [16]. Moreover, transgenic rice plants that over-expressed miR393 and miR396c were more sensitive to salt stress [17, 18]. For a large number of plant species, it is becoming increasingly evident that miRNAs play an important role in plant salt stress. Therefore, more studies on miRNA expression in response to salt stress in plants are required. Furthermore, more research at the genome level using high-throughput sequencing methodologies should facilitate future discovery of additional alkaline-salt stress responsive miRNAs in plants.

Flax (*Linum usitatissimum*) is a member of the genus *Linum* in the family Linaceae and is grown as a food and fiber crop worldwide. Attempts have been made to grow flax in saline-alkaline soil in order to avoid competition for land with other food crops with limited success. Achieving better yields would greatly improve flax fiber supply and foster sustainable development practices in the flax industry. Therefore, using different strategies, flax breeders have made great efforts to develop a salt tolerant flax cultivar [19, 20]. However, the successful cultivation of salt tolerant flax varieties has not yet been reported. Fortunately, the recent release of the flax genome sequence has furthered understanding of transcriptional level molecular mechanisms of flax adaptation to saline-alkaline stress [21]. Moreover, digital gene expression has recently resulted in identification of several differentially expressed genes in flax under saline-alkaline stress [22]. However, miRNA expression profiling and miRNA targeted genes during saline-alkaline stress in flax have yet to be elucidated. To date, only three reports have been published focusing on flax miRNAs, but they all employed bioinformatics tools to predict flax miRNAs [23–25].

To provide further insights into the role of miRNAs in flax tolerance to saline, alkaline, and saline-alkaline stresses, small RNA and degradome high-throughput sequencing was conducted to analyze samples of flax seedlings grown under alkaline-salt stress (AS2), neutral salt stress (NSS), alkaline stress (AS), and under control conditions (CK). In this study, flax miRNAs, their target genes, functional annotations, and gene expression patterns were revealed under saline, alkaline, and saline-alkaline stresses. These findings should enhance the understanding of regulatory mechanisms involving flax miRNAs expression under saline, alkaline, and saline-alkaline stresses and provide a foundation for future studies to determine the specific functions of these miRNAs. This study is the first report in which small RNA (sRNA) libraries have been constructed and sequenced to identify saline, alkaline, and saline-alkaline tolerance miRNAs in flax.

## Result

### Characterization of Lus-miRNAs from deep sequencing of flax sRNA libraries

To identify saline, alkaline, and saline-alkaline responsive miRNAs in flax, four small RNA libraries from flax seedlings treated with AS, AS2, NSS, and water (control) were constructed. Solexa, a high throughput sequencing technology, was employed to sequence these libraries, leading to generation of over 26.5 million clean reads from four libraries. All clean reads were obtained after removal of adapter, insert, and polyA sequences, as well as removal of sequences of RNAs shorter than 18 nt in length (Table 1). Ultimately, over 2.7 million unique sRNAs from four libraries were mapped to the flax genome published in 2012 [21].

**Table 1:** Summary of data cleaning of MicroRNA-seq

Library	Raw reads	Adaptors removed	Sequences < 18 nt removed	Clean reads	Total sRNAs mapped to Genome	Unique sRNAs mapped to Genome
CK	31,675,979	136,110	58,807	31,309,326	24,393,572	3,486,958
AS	29,867,252	65,980	105,870	29,526,585	19,793,866	3,488,851
AS2	27,379,453	92,632	593,274	26,536,356	18,749,253	2,767,309
NSS	28,449,740	114,587	64,740	28,105,246	22,212,514	3,058,192

*Abbreviations*: *AS* Alkaline stress, *AS2* Alkaline-salt stress, *CK* Control, *Lus Linum usitatissimum*, *NSS* Neutral salt stress

**Fig. 1:**
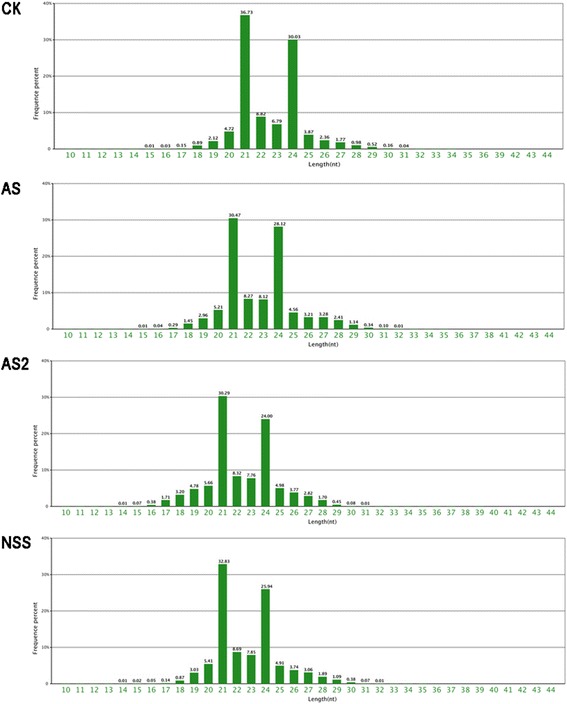
Summary of the read length distribution of small RNAs. The distributions of the total reads are shown as percentages

The size distribution of all sRNAs was found to be diverse, ranging from 18–30 nt in length, with the majority measuring 19–25 nt in length (Fig. 1). The sRNAs of 21 nt and 24 nt formed two major classes within the total sRNA. In addition, analysis of the first nucleotide of 18–30 nt sRNAs indicates that many sRNAs possess a uridine (U) at their 5’ ends. Most of these sRNAs are 21 nt and 22 nt long, with the 21 nt length predominating (Additional file 1). Similar to observations in other plants, most miRNAs here were of 21 and 22 nt in length and possessed a 5’ uridine, which is one of the important characteristic features of miRNAs.

High throughput sequencing can be used to verify a large number of known miRNAs and novel specific miRNAs in organisms. From four sRNA libraries in this study, CK, AS, AS2, and NSS libraries, we first identified 118, 119, 122, and 120 known Lus-miRNAs, respectively. These were assigned to 23 conserved miRNA families after comparing our libraries with known miRNAs from flax and other plant species using miRBase 19.0 (http://www.mirbase.org/). Bioinformatics analysis of the sequencing data, based on the criteria of novel miRNA annotations developed by Meyers [26], resulted in identification of 233, 213, 211, and 212 potential novel Lus-miRNAs in the CK, AS, AS2, and NSS libraries, respectively (Additional file 2).

### Discovery of miRNAs responsive to saline, alkaline, and saline-alkaline stresses in flax

To identify Lus-miRNAs responsive to saline, alkaline, and saline-alkaline stresses, differentially expressed miRNAs in each sample were compared to the control. A false discovery rate (FDR) <0.001 and an absolute threshold value of the log2 ratio fold-change >1 were used to determine the statistical significance of relative miRNA abundance values. There were 101, 103, and 101 differentially expressed known miRNAs in the AS, AS2, and NSS libraries, respectively (Additional file 3). Of these, 32, 37, and 14 were up-regulated and 69, 66, and 87 were down-regulated in the libraries, respectively. Among these, 2, 19, and 13 miRNAs exhibited very high expression differences in their respective libraries, relative to the control (Table 2).

**Table 2:** Summary of significant differential expressed genes known miRNA under saline, alkaline, and saline-alkaline stresses

MiR-name	Fold-change(log2)			Target	Anotation
	AS	ASS	NSS		
lus-miR159c	-	-	−1.0931792↓	Lus10036103[a], Lus10016550, Lus10027189, Lus10017946, Lus10008685[a], Lus10028176, Lus10013688, Lus10035275, Lus10026142[a], Lus10009780, Lus10026787[a]	Myb domain protein, Mitochondrial transcription termination factor family protein, Transcription regulators
lus-miR160a/e/f	-	1.20840613↑	−3.66411747↓	Lus10024753, Lus10024754, Lus10023519, Lus10019940, Lus10026510, Lus10016090, Lus10040403, Lus10009770, Lus10021467	Auxin response factor
lus-miR160b/d	-	1.20719184↑	−3.66660396↓		
lus-miR160j	-	1.20846291↑			
lus-miR160h/i	-	1.20203167↑	−3.69976116↓		
lus-miR169c	-	−1.13471672↓	-	Lus10017991, Lus10041986,	Jasmonate-zim-domain protein, GRAS family transcription factor
lus-miR169e/i	-	−1.05752449↓	-		
lus-miR171j	-	-	−1.02479034↓	Lus10024029, Lus10041721, Lus10004353, Lus10028934	GRAS family transcription factor
lus-miR319a	-	-	−1.46989392↓	Lus10036103[a], Lus10008685[a], Lus10026142[a], Lus10026787[a]	Myb domain protein
lus-miR393a/c	-	1.12123117↑	-	Lus10031991, Lus10035160	Auxin signaling F-box
lus-miR393b/d		1.00414957↑	-		
lus-miR394a	-	2.65759041↑	−3.63085489↓	Lus10000973, Lus10029731, Lus10011354, Lus10022009, Lus10028656, Lus10028656, Lus10006975, Lus10015775, Lus10001312, Lus10003117, Lus10037030,	S-adenosyl-L-methionine-dependent methyltransferases superfamily protein, Galactose oxidase/kelch repeat superfamily protein, Signal transduction histidine kinase, FGGY family of carbohydrate kinase, Jasmonate-zim-domain protein
lus-miR394b	-	2.77702791↑	−3.82176614↓		
lus-miR398a	2.51091239↑	2.69813582↑	-	-	-
lus-miR408a	1.71297796↑	1.63630636↑	-	Lus10018938, Lus10020012, Lus10028640, Lus10028641	Plantacyanin, Chloroplast import apparatus

*Abbreviations*: *AS* Alkaline stress, *AS2* Alkaline-salt stress, *CK* Control, *Lus Linum usitatissimum*, *NSS* Neutral salt stress, ↑, Upregulated; ↓, Downregulated; [a], the same target genes of lus-miR159c and lus-miR319a

Of particular interest, 66, 66, and 56 novel miRNAs were differentially expressed and of these, 28, 27, and 16 were up-regulated and 38, 39, and 40 were down-regulated in the AS, AS2, and NSS libraries, respectively (Additional file 3). Of these, 38, 34, and 34 were highly differentially expressed (Additional file 4). Moreover, several miRNAs were significantly, differentially expressed between two separate libraries. For example, lus-miR398a and lus-miR408a were significantly differentially expressed between AS and AS2, whereas lus-miR160 and lus-miR394 were significantly differentially expressed between AS2 and NSS.

The expression levels of known miRNAs and novel miRNAs in all samples are listed in Additional file 5. The results revealed that two known Lus-miRNAs (lus-miR399f and lus-miR399g) and five novel Lus-miRNAs (novel_mir_147, novel_mir_150, novel_mir_27, novel_mir_2, novel_mir_45) were found only in AS, AS2 and NSS, but not in CK, suggesting they were probably induced by saline, alkaline, and saline-alkaline stresses.

### The expression patterns of all known and novel miRNAs under saline, alkaline, and saline-alkaline stresses in flax

**Fig. 2:**
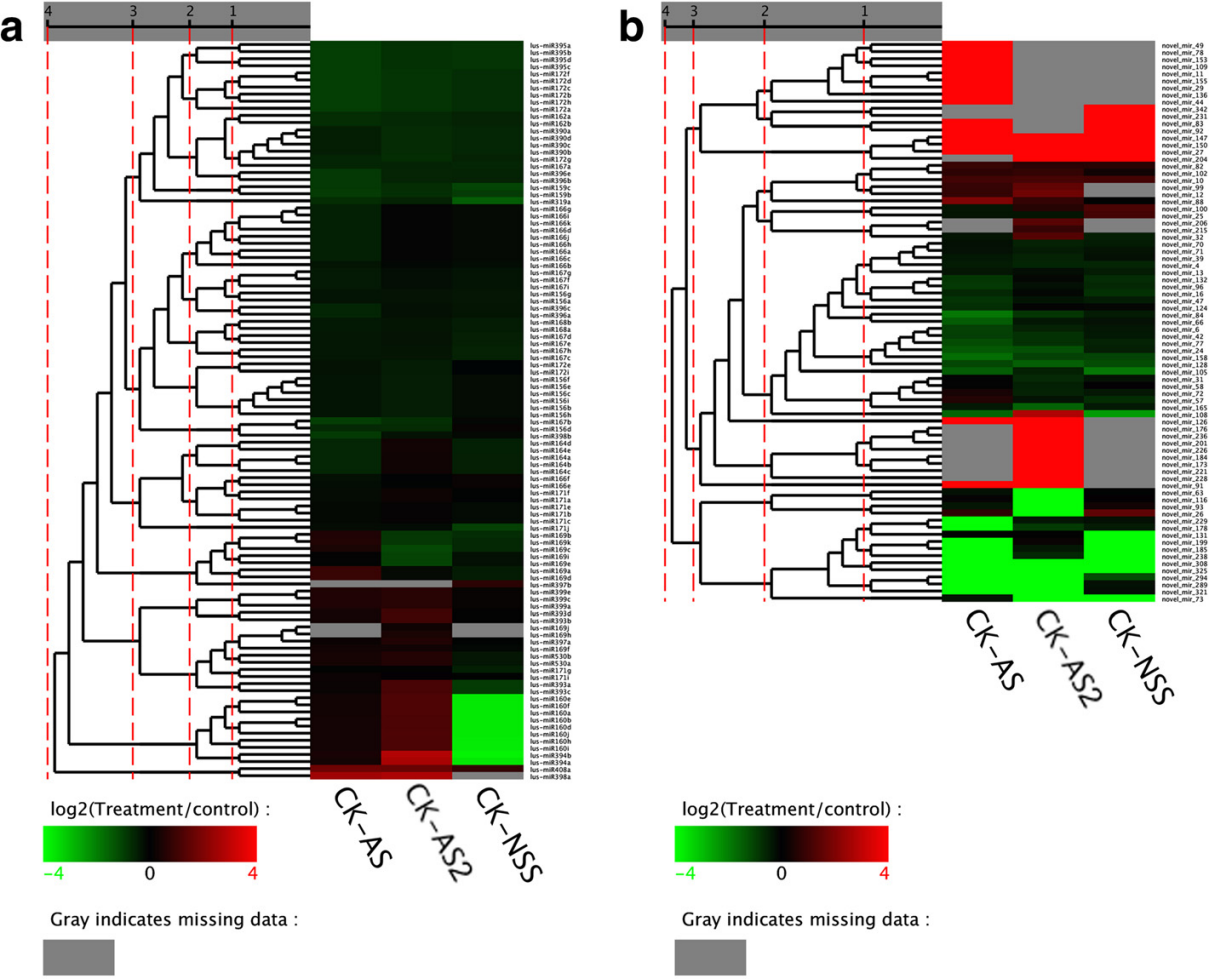
Cluster analyses of known miRNAs and novel miRNAs. Each line refers to data from one gene. The color bar represents the log_2_RPKM and ranges from green to red. Red indicates that the miRNA has a higher expression level in treated sample; green indicates that the miRNA has higher expression in the control sample and gray indicates that the miRNA has no expression in at least one sample; dotted line indicates that all differentially expressed miRNAs are clustered all in one after four rounds of cluster.

The expression patterns of all known and novel miRNAs identified were profiled based on their sequencing results. Most of the known and the novel miRNAs showed various degrees of expression under saline, alkaline, and saline-alkaline stresses as compared to the control, with the log2 values (Treatment/Control) of the fold changes falling between -4 and 4 (Fig. 2, Additional file 3). Of the 23 known miRNA families, 2 (lus-miR408, lus-miR530) and 5 (lus-miR160, lus-miR393, lus-miR394, lus-miR398, lus-miR408) were up-regulated significantly in AS and AS2, respectively. 1 (lus-miR169) and 5 families (lus-miR159, lus-miR160, lus-miR171, lus-miR319, lus-miR394) were down-regulated significantly in AS2 and NSS, respectively (Fig. 2a, Additional file 3). Of the novel miRNAs, 18, 20, and 12 were significantly up-regulated, while 20, 14, and 12 were significantly down-regulated in AS, AS2, and NSS libraries, respectively (Fig. 2b, Additional file 3). These results suggest that these miRNAs might have coordinating functions in response to saline, alkaline, and saline-alkaline stresses in flax.

Our data also showed that some members of the same miRNA family exhibited different expression patterns under saline, alkaline, and saline-alkaline stresses in flax (Fig. 2). For example, Lus-miR171g is up-regulated in AS but down-regulated in AS2 and NSS, while Lus-miR171i is up-regulated in AS and NSS but down-regulated in AS2. Although these results await further confirmation using other molecular techniques, together they suggest that miRNA members from different families, as well as different members from the same family, may have variable response patterns to saline, alkaline, and saline-alkaline stresses.

### Prediction and annotation of miRNA target genes

To further understand the potential functions of the known and novel salt-responsive miRNAs identified in this work, their putative target genes were predicted using the psRNA Target program (http://plantgrn.noble.org/psRNATarget/). 17 differentially expressed known miRNAs and 36 differentially expressed novel miRNAs with high abundance were selected and used to predict putative target genes (Table 2, Additional file 6). Among the 36 novel Lus-miRNAs, 22 had multiple target genes, as exemplified by the novel_mir_231 Lus-miRNA with 261 target genes, indicating these Lus-miRNAs might possess comprehensive functions in flax. Interestingly, while different members of a given miRNA family may target the same genes, even members of diverse miRNA families may also share common target genes. For example, both lus-miR169e and lus-miR169i can target genes encoding jasmonate-zim-domain protein 3, which indicates that they are functionally conservative members within one family, with similar results observed for lus-miR394a and lus-miR394b. However, members of these distinct families also share a gene target belonging to the FGGY family of carbohydrate kinase. Furthermore, lus-miR159c and lus-miR319a can target the same gene encoding a Myb domain protein, which means their functions may be similar under saline stress in flax (Table 2).

To evaluate the potential functions of these miRNA target genes, GO analysis was used [27]. The miRNA target genes were categorized according to biological process, cellular component, and molecular function (Additional files 7 and 8). The miRNA predicted targets in AS showed enrichment in GO terms in the biological process category, while no enrichment in GO terms was observed in the cellular component and molecular function categories. The results reveal that these target genes possess functions involved in response to stimuli, signaling, catalytic activity, and their expression is significantly altered by saline, alkaline and saline-alkaline stresses, in comparison to genes in CK as a whole.

### MiRNA targets verified by degradome sequencing

To further understand the role of miRNA in saline, alkaline and saline-alkaline stresses regulation in flax, degradome sequencing of flax was used to identify miRNA targets (Additional file 9). Although a large number of transcripts exhibited expression changes under saline, alkaline and saline-alkaline stresses, 29 miRNA-target pairs showed inverse expression pattern changes when the results from miRNA profiling, degradome sequencing, and transcriptome profiling from our previous study were compared (Table 3) [22]. These results indicate that these miRNAs and target genes may play important opposing roles in the response to saline, alkaline and saline-alkaline in flax.

**Table 3:** Complementary expressions between miRNAs and their targets in flax under saline, alkaline, and saline-alkaline stresses

MiRNAs	Small RNA sequencing			Target gene	Annotation	Degradome sequencing			DGE sequencing		
	AS	AS2	NSS			Position	Lignment score	Category	AS	AS2	NSS
lus-miR156g/a	Down	Down	Down	Lus10000257	Tetratricopeptide repeat (TPR)-like superfamily protein	761	4.5	4	Up	Up	Up
lus-miR159b	Down	Down	Down	Lus10010495	Cystatin/monellin superfamily protein	335	4.5	3	Up	Up	Up
lus-miR160a/b/d/e/f/h/i/j	Up	Up	Down	Lus10041268	Transducin/WD40 repeat-like superfamily protein	1014	4	2	Down	Down	Up
lus-miR162a/b	Down	Down	Down	Lus10015483	Heat shock protein 70 (Hsp 70) family protein	1243	4.5	2	Up	Up	Up
lus-miR164a/b/c/d/e	Down	Up	Down	Lus10006635	ARM repeat superfamily protein	153	4	4	Up	Down	Up
lus-miR166a/c/d/g/h/j	Down	Down	Down	Lus10020493	Pathogenesis-related gene 1	220	4	2	Up	Up	Up
lus-miR167a	Down	Down	Down	Lus10014324	G-box binding factor 1	679	3.5	2	Up	Up	Up
lus-miR168a/b	Down	Down	Down	Lus10000074	Methionine gamma-lyase	469	4.5	4	Up	Up	Up
lus-miR169a/d	Up	Down	Down	Lus10014674	Transducin/WD40 repeat-like superfamily protein	638	4	4	Down	Up	Up
lus-miR169e/i	Down	Down	Down	Lus10006846	Profilin 5	96	4.5	4	Up	Up	Up
lus-miR169g/l	Up	Down	Up	Lus10030904	Alpha/beta-Hydrolases superfamily protein	755	4.5	4	Down	Up	Down
lus-miR171b/c/e	Down	Down	Down	Lus10009876	UDP-glucosyl transferase 85A3	952	4	4	Up	Up	Up
lus-miR171d	Up	Down	Down	Lus10017991	Jasmonate-zim-domain protein 3	594	4.5	2	Down	Up	Up
lus-miR172a/b/c/d/f/h	Down	Down	Down	Lus10001322	Deoxyxylulose-5-phosphate synthase	1796	4	4	Up	Up	Up
lus-miR319b	Down	Down	Down	Lus10009442	O-methyltransferase family protein	502	4	4	Up	Up	Up
lus-miR390a/b/c/d	Down	Down	Down	Lus10015906	Purine permease 3	857	4.5	4	Up	Up	Up
lus-miR393a/b/c/d	Up	Up	Down	Lus10040438	F-box family protein	1620	3.5	4	Down	Down	Up
lus-miR394a/b	Up	Up	Down	Lus10018337	Pyruvate dehydrogenase kinase	536	4.5	2	Down	Down	Up
lus-miR395a/b/c/d	Down	Down	Down	Lus10006629	ATP sulfurylase 1	339	2.5	0	Up	Up	Up
lus-miR396a/c	Down	Down	Down	Lus10001321	Xylose isomerase family protein	648	3.5	2	Up	Up	Up
lus-miR397a	Up	Up	Down	Lus10004434	REF4-related 1	1875	4.5	4	Down	Down	Up
lus-miR397b	Down	Up	Up	Lus10001002	3-deoxy-d-arabino-heptulosonate 7-phosphate synthase	581	4.5	4	Up	Down	Down
lus-miR398a	Up	Up	Up	Lus10016155	Copper/zinc superoxide dismutase 2	449	4	0	Down	Down	Down
lus-miR398b/c/d/e	Down	Down	Down	Lus10003315	Myosin family protein with Dil domain	4406	4.5	2	Up	Up	Up
lus-miR399b/d	Down	Up	Down	Lus10019360	Trigalactosyldiacylglycerol2	323	4.5	4	Up	Down	Up
lus-miR399f/g	Up	Up	Up	Lus10003060	Cofactor-independent phosphoglycerate mutase	1097	4	4	Down	Down	Down
lus-miR408a	Up	Up	Up	Lus10003138	Cyclophilin 20-2	860	4.5	2	Down	Down	Down
lus-miR530a/b	Up	Up	Down	Lus10001902	WRKY family transcription factor	490	4.5	2	Down	Down	Up
lus-miR828a	Down	Up	Up	Lus10013640	Ribosomal protein L3 family protein	570	4.5	4	Up	Down	Down

*Abbreviations*: *ARM* Armadillo, *AS* Alkaline stress, *AS2* Alkaline-salt stress, *ATP* Adenosine-triphosphate, *CK* Control, *DGE* Digital gene expression, *Hsp* Heat shock protein, *Lus Linum usitatissimum*, *MiRNA* MicroRNA, *NSS* Neutral salt stress, *REF* Reduced epidermal fluorescenc, *TPR* Tetratricopeptide repeat, *UDP* Uridine diphosphate

### qRT-PCR analysis of miRNAs under saline, alkaline, and saline-alkaline stresses in flax

**Fig. 3:**
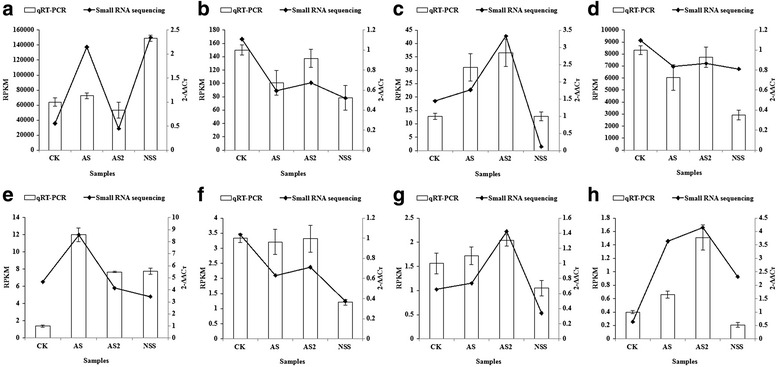
Comparison of the miRNA expression profiles determined by quantitative real-time RT-PCR (qRT-PCR) and deep sequencing. Bars represent the standard deviations of three replicates. **a** lus-miR156b; **b** lus-miR159c; **c** lus-miR160a; **d** lus-miR168a; **e** lus-miR169a; **f** lus-miR319a; **g** lus-miR393a; **h** lus-miR398a

To confirm the accuracy and reliability of the miRNA-seq results, the same RNA preparation used for Solexa sequencing was used to prepare samples for the qRT-PCR assay. In this study, eight miRNAs (lus-miR156b, lus-miR159c, lus-miR160a, lus-miR168a, lus-miR169a, lus-miR319a, lus-miR393a and lus-miR398a) were randomly selected for analysis of expression levels under saline, alkaline, and saline-alkaline stresses using actin as the internal reference gene (Fig. 3). Results showed that the expression changes of these miRNAs, as determined by qRT-PCR, followed similar trends observed using sequencing results. These results suggest that differentially expressed flax miRNAs had been successfully and accurately identified under saline, alkaline, and saline-alkaline stresses using Solexa sequencing. Of note, the abundance of miR159c, miR168a, and miR319a were lower under saline-alkaline stress.

## Discussion

High throughput sequencing technology has been extensively applied to small RNA research. MiRNAs, as regulators of target genes, have been reported to play major roles in a plant’s response to abiotic stresses, including dehydration, freezing, salinity, and alkalinity [28]. Many miRNAs involved in the high-salinity stress response in plants have been identified [29, 30]; however, little research on a genome-wide scale has focused on the saline, alkaline, and saline-alkaline responsive miRNAs in flax. In the present study, miRNAs were identified and characterized from flax under saline, alkaline, and saline-alkaline stresess using experimental characterization of sRNAs. This work will provide new information to facilitate further research into the functions, biological pathways, and evolution of flax sRNA and its target genes.

In this study, we constructed four sRNA cDNA libraries from flax seedlings treated with AS, AS2, NSS, and CK. Subsequently, 124 known miRNAs belonging to 23 conserved miRNA families and 394 novel miRNAs were identified after sequencing and analysis of the sRNAs of flax. Analysis of the predicted targets of the miRNAs using the GO and KEGG databases indicated that a range of metabolic pathways and biological processes known to be associated with salt stress were up-regulated in flax treated with salt. Most of the miRNAs that were obtained in our library have a 5’-U, as has been reported in other plants, which is in accordance with the known structures of the mature miRNAs [31]. The results indicated the presence of a range of sRNAs, of lengths 14–32 nt in flax, with most of the unique sequence reads of 24 nt in length with 21 nt length reads next in predominance. This observation is in agreement with previous reports for grapevine and soybean [32, 33], as well as with results indicating that the most common sRNAs in celery and maize were 24 nt in length [34, 35]. However, these results differ from results reported for Chinese cabbage and foxtail millet. Some plant species, including *Arabidopsis thaliana*, had been shown to contain substantially more 24 nt sRNAs than 21 nt sRNAs [36]; on the other hand, sRNAs populations with more members of length 21 nt than 24 nt were reported in *Brassica juncea* and in Japanese apricot with imperfect flower buds [37, 38]. Taken together, all of these results suggest that some differences might exist in sRNA biogenesis pathways between various plant species.

Many miRNAs with a wide range of expression levels were found in the AS, AS2, NSS, and CK libraries. The most abundantly expressed miRNA family across the four libraries was miR156, specifically including miR156b, miR156c, miR156e, miR156f, miR156h, and miR156i, as was also observed in *Leymus chinensis* (Additional file 6) [39]. Some miRNAs were differentially expressed between the stress libraries and control library (Additional file 3). There were 2, 19, and 13 highly differentially expressed miRNAs in AS, ASS, and NSS, as compared to CK, respectively. Two miRNAs (lus-miR398a and lus-miR408a) were greatly up-regulated in AS and AS2 as compared to CK, in opposition to the results of pea plants subjected to drought stress [40]. Expression of ten miRNAs (lus-miR160a, lus-miR160b, lus-miR160d, lus-miR160e, lus-miR160f, lus-miR160h, lus-miR160i, lus-miR160j, lus-miR394a and lus-miR394b) were significantly altered in AS2 and NSS as compared to CK, however, these miRNAs were significantly up-regulated in AS2 and significantly down-regulated in NSS. The results indicated that these lus-miRNAs exhibit different functions in response to AS2 and NSS in flax. In agreement with our results, previous studies have consistently demonstrated that miR394 was responsive to stress conditions, including salt and drought stress [41]. However, miR160 only previously had been reported to play an important role in plant development, not in stress responses, as shown in this work [42].

Our libraries have facilitated the identification of a large number of conserved saline, alkaline, and saline-alkaline responsive lus-miRNAs, including lus-miR156, lus-miR159, lus-miR160, lus-miR162, lus-miR164, lus-miR166, lus-miR167, lus-miR168, lus-miR169, lus-miR171, lus-miR172, lus-miR319, lus-miR390, lus-miR393, lus-miR394, lus-miR395, lus-miR396, lus-miR397, lus-miR398, lus-miR399, lus-miR408, lus-miR503, and lus-miR828, some of which were confirmed here using qRT-PCR (Fig. 3). Several differentially regulated miRNAs have been identified in salt-stressed plants. Our results agree with results in a previous study involving *Arabidopsis thaliana* [43], *Zea mays* [44], *Vigna unguiculata* [45], *Populus trichocarpa* [46], *Populus tremula* [13], *Oryza sativa* [47, 48], in which 22 salt-responsive miRNAs (miR156, miR159, miR160, miR162, miR164, miR166, miR167, miR168, miR169, miR170/miR 171, miR172, miR319, miR390, miR393, miR394, miR395, miR396, miR397, miR398, miR399, miR408 and miR530) were reported to be involved in the high salinity stress response (Table 4). In *Arabidopsis thaliana*, miR156, miR159, miR167, miR168, miR171, miR319, miR393, miR394, miR396, and miR397 were all up-regulated in response to salt stress, whereas miR398 was down-regulated [14, 43]. Furthermore, miR169g and miR169n were also reported to be induced by high salinity [49]. Recently, a study of maize roots using miRNA microarray hybridization indicated that members of the miR156, miR164, miR167, and miR396 families were down-regulated by salt shock, whereas miR162 and miR168 were up-regulated [44]. The expression of miR389, miR400, miR402, miR403, and miR407a were inhibited by salt, cold, dehydration, and abscisic acid (ABA) in *Arabidopsis* [14], while these miRNAs and their variants were not detected in flax.

**Table 4:** MicroRNAs responsive to neutral saline stress (NaCl) in diverse plant species

MiR-name	Plant species	Refs
miR156	Lus↑&↓, Zma↓, Ath↑, Vun↑	43,44,49
miR159	Lus↓^a^, Ath↑, Osa↓	43,48
miR160	Lus↓^a^, Osa↓, Vun↑	43,48
miR162	Lus↓, Zma↑, Vun↑	43,44
miR164	Lus↓, Zma↓	44
miR166	Lus↑&↓	
miR167	Lus↓, Zma↓, Ath↑	43,44
miR168	Lus↓, Zma↑, Ath↑, Pte↑, Vun↑	13,16,44,49
miR169	Lus↓, Zma↑, Ath↑, Pte↓, Osa↑, Vun↑	13,16,43,49
miR170/miR171	Lus↑&↓^a^, Ath↑, Ptc↓	43,52
miR172	Lus↑&↓	
miR319	Lus↓^a^, Ath↑, Osa↓	43,48
miR390	Lus↓	
miR393	Lus↓, Ath↑, Ptc↑, Osa↓	43,49
miR394	Lus↓^a^, Ath↑, Osa↓	43,48
miR395	Lus↓, Zma↑, Pte↑	13,44
miR396	Lus↓, Zma↓, Ath↑, Osa↓	43,44,49
miR397	Lus↑, Ath↑	14
miR398	Lus↓, Ath↓, Pte↑	13,43
miR399	Lus↑, Pte↑	13
miR408	Lus↑, Vun↑	49
miR530	Lus↓, Ptc↓, Osa↓	48,52

*Abbreviations*: *Ath Arabidopsis thaliana*, *Lus Linum usitatissimum*, *MiR* MicroRNA, *Ptc Populus trichocarpa*, *Pte Populus tremula*, *Osa Oryza sativa*, *Vun Vigna unguiculata*; *Zma Zea mays*, ↑, Upregulated; ↓, Downregulated; ↑&↓, Some members were upregulated, and some were downregulated

^a^Significant differential expressed known miRNA in flax

In this study, target genes for miRNAs that were differentially expressed in the four libraries were identified by searching for corresponding plant miRNA target sites, which are predominantly located in open reading frames. Many antioxidant enzyme and transcription factors have been predicted to be targets of conserved, flax-specific miRNAs (Table 3). Some of these proteins have been well-studied and their roles in salt tolerance or the stress response have been established. Previous studies have demonstrated that miR398 family members are associated with high salt stress [13]. From our target prediction analysis, the miR398a-targeted gene codes for a copper/zinc superoxide dismutase (CuZnSOD, EC l.15.1.1), known to be important scavengers of reactive oxygen species (ROS) to protect cells from damage. Recent studies have also demonstrated that this protein plays significant roles in salt stress response pathways. These results are in agreement with our data for miR398 [50, 51], suggesting significant involvement of this miRNA and its target in the salt stress response in plants.

In this work, the salt-responsive miR530 has been shown to explicitly target WRKY family transcription factors (TFs). This is in agreement with previous findings showing that plant-specific WRKY TFs are involved in stress responses such as cold, high salinity or drought, as well as in abscisic acid signaling. A parallel study reported that WRKY TFs act in response to salt stress in many plants, including rice [52], maize [53], and cotton [54]. However, only one paper has focused on miR530, demonstrating that the target gene of miR530 was KNAT [55], which regulates inflorescence architecture in *Arabidopsis* [56]. Therefore, the relationship between miR530 and salt stress should be given more attention. Meanwhile, there are several miRNAs without identified target genes; these results could be the result of inaccurate target predictions, or these might be low-abundance miRNAs with limited or no activity. It is also possible that miRNAs might exist that have no targets. Nevertheless, the KO and GO analyses revealed that many of the genes targeted by miRNAs in flax are related to salt stress, supporting the hypothesis that miRNAs play an important role in the response of flax to salinity. Greater understanding of these miRNAs and their targets should facilitate future development of flax with greater resistance to salt stress.

## Conclusions

Four small RNA libraries and one degradome library were constructed under saline, alkaline, and saline-alkaline stresses in flax. By using high-throughput sequencing, the miRNAs profile of flax was investigated to illustrate the miRNAs expression differences among AS2, NSS and AS. Many known Lus-miRNAs and potential novel Lus-miRNAs were identified in the CK, AS, AS2, NSS libraries, respectively. After assessment of differential expression profiles, 17 known Lus-miRNAs and 36 novel Lus-miRNAs were selected and used to predict putative target genes. Gene ontology term enrichment analysis revealed target genes that were involved in responses to stimuli, including signaling and catalytic activity. Eight Lus-miRNAs were selected for analysis using qRT-PCR to confirm the accuracy and reliability of the miRNA-seq results. Degradome sequencing and transcriptome profiling showed that expression of 29 miRNA-target pairs displayed inverse expression patterns under saline, alkaline, and saline-alkaline stresses. Identification and characterization of flax miRNAs, their target genes, functional annotations, and gene expression patterns are reported in this work. These findings will enhance our understanding of flax miRNA regulatory mechanisms under saline, alkaline, and saline-alkaline stresses and provide a foundation for future elucidation of the specific functions of these miRNAs.

## Methods

### Plant materials and stress treatments

The fiber flax plant cultivar used in this study, Heiya No. 19, was obtained from the Industrial Crops Institute, Heilongjiang Academy of Agricultural Sciences (Harbin, P.R.China). Flax seeds were grown on sterilized vermiculite in cups. All plants were cultivated in climate chambers at 22 °C day/18 °C night with a 16 h day/8 h night photoperiod cycle, 70 % relative humidity, and a light intensity of 3000 lx. The plants were irrigated with one-half strength Murashige and Skoog medium every 3 days.

The stress treatment was the same as previously described [22]. For the treatments, the 3-week-old seedlings showing appropriate growth states were exposed to alkaline-salt stress (AS2, 25 mM Na_2_CO_3_, pH 11.6), neutral salt stress (NSS, 50 mM NaCl), and alkaline stress (AS, NaOH, pH 11.6), respectively. In parallel, the same numbers of seedlings were transferred to distilled water as a control (CK). After exposing the seedlings to stress solutions for 18 h, whole seedlings were harvested, frozen immediately in liquid nitrogen, and stored at -80 °C before use. The control plants were also harvested and frozen at the same time. There were more than ten seedlings in each sample.

### Construction and sequencing of small RNA libraries

Total RNA was extracted from flax using Trizol Reagent (Invitrogen, USA), following the manufacturer’s instructions. Total RNA quantity and purity were assayed with the NanoDrop 2000 spectrophotometer (Thermo Scientific, USA) at 260/280 nm (ratios were between 1.8 and 2.0). After assessing RNA integrity using 2 % agarose gel electrophoresis, four sRNA libraries were constructed using RNA extracted from the four different treatments. The four libraries were sequenced using Solexa sequencing (Illumina, USA) at the Beijing Genomics Institute (BGI, Shenzhen, China).

### Bioinformatic analysis of miRNAs

The 49 nt sequence reads from HiSeq sequencing were subjected to data cleaning analysis to remove low quality sequence tags and 5’ adaptor contaminants from the reads, leaving clean reads for subsequent analysis. Next, the length distribution of the clean reads for the various samples was summarized. Next, alignment of small RNAs to the miRNAs precursor of the corresponding flax species was performed using miRBase to obtain the miRNAs count using the following detailed criteria. First, high stringency alignment of reads to the miRNAs precursor was performed in miRBase with no mismatches. Second, based on the first criteria, the reads were next aligned to the mature miRNAs in miRBase with at least a 16 nt overlap allowing offsets. Those miRNAs satisfying both criteria were counted to measure the expression of identified miRNAs and were next analyzed to determine the base bias for the first position of identified miRNAs of certain lengths and the base bias for each position of all identified miRNAs, respectively. The novel miRNA was de novo identified by mapping to the genome and predicting loci using Mireap.

To identify differences in miRNAs expression levels under salt stress, the number of reads for each identified miRNA was normalized against the total number of reads in the corresponding library. Comparison of the known miRNAs expression between two samples allowed identification of differentially expressed miRNAs. Comparison of the expression of miRNAs between two samples was visualized by plotting a Log2-ratio figure showing clustering of miRNAs with similar expression patterns in both samples.

### Prediction of miRNA targets

After identifying miRNAs with expression patterns that reflect responses to salt stress, putative targets of known and novel miRNAs were predicted using psRNATarget (http://plantgrn.noble.org/psRNATarget/) with default parameters using flax genome settings (http://phytozome.jgi.doe.gov/pz/portal.html). MiRNA targets were next further validated using degradome sequencing. To further investigate the biological functions of miRNAs in flax, the predicted target genes were used to annotate their functions and pathways using the Gene Ontology database (GO, http://www.geneontology.org/). The GO terms corresponding to the target genes were annotated according to their biological processes, molecular functions or involvement as cellular components using Blast2GO.

### Degradome sequencing sampling/procedures

By using Illumina Hiseq 2000 sequencing system, degradome sequencing takes SE50 sequencing strategy and produces 49 nt raw reads. The 3’ adaptor will be trimmed before bioinformatics analysis to get real degradome fragments whose length between 20 and 21 nt. After preprocssing, clean tags are generated and stored. Classify clean tags by the alignment to database and remove the ncRNAs. At last, identify the miRNA-mRNA pairs by mapping to reference genes.

The tags mapped to cDNA_sense were used to predict cleavage sites. The specific sites at which a miRNA will cleave gene. Cleavage sites at different positions of one gene are different cleavage sites. The specific miRNA-mRNA pair at cleavage site. Cleavages induced by different miRNAs sequences are different cleavage events, no matter if they are at the same cleavage site. The height of the degradome peak at each occupied transcript position is placed into one of five possible categories: 0, >1 raw reads at the position, abundance at position is equal to the maximum on the transcript, and there is only one maximum on the transcript; 1, >1 raw reads at the position, abundance at position is equal to the maximum on the transcript, and there is more than one maximum position on the transcript; 2, >1 raw reads at the position, abundance at position is less than maximum but higher than the median for the transcript; 3, >1 raw reads at the position, abundance at position is equal to or less than the median for the transcript; 4, Only 1 raw read at the position.

### Detection of miRNA expression using qRT-PCR to validate sequencing results

To validate the results from the bioinformatics-based analysis, stem-loop real-time quantitative RT-PCR was performed. The qRT-PCR primers were designed from miRNA sequences determined above and the reverse transcription was carried out using the RNA from the same four flax libraries (Additional file 10). qRT-PCR was performed using an ABI 7500 Real-Time PCR System (Applied Biosystems, USA) using SYBR Green I (TOYOBO) with the following program: 94 °C for 30s, followed by 40 cycles of 94 °C for 15 s, 60 °C for 15 s, and 72 °C for 45 s. At the end of PCR reaction, a melting curve was determined. All reactions were conducted at least three times using U6 as an internal control. Three technical replicates were used for qRT-PCR. The relative expression of the miRNA was calculated using the 2^-ΔΔCt^ method. Statistical tests for qRT-PCR comparisons and small RNA seq was performed by dual axis mapping method of Excel.

## Additional files

Additional file 1: First nucleotide bias of 18–30 nt sRNA tags. (TIF 4533 kb) Additional file 2: Summary of known miRNA and novel miRNA in each sample. (XLS 19 kb) Additional file 3: Summary of intersample differential expression known miRNA and novel miRNA. (XLS 92 kb) Additional file 4: Summary of significant differential expressed novel miRNA. (XLS 32 kb) Additional file 5: Summary of known miRNA and novel miRNA expression level in all samples. (XLS 81 kb) Additional file 6: Identified targets of significant differential expressed novel miRNAs in flax. (XLS 22 kb) Additional file 7: GO ontology statistics of target genes of differential expressed known miRNAs and novel miRNAs. (PDF 275 kb) Additional file 8: GO ontology statistics of targets of known miRNAs and novel miRNAs having differential expression. (XLS 20 kb) Additional file 9: Summary of data cleaning of degradome sequencing. (XLS 58 kb) Additional file 10: The primers used in this study. (XLS 8 kb)

## Abbreviations

ABA: abscisic acid
AS: alkaline stress
AS2: alkaline-salt stress
BGI: Beijing Genomics Institute
CK: control
CuZnSOD: copper/zinc superoxide dismutase
FDR: false discovery rate
GO: gene ontology
KEGG: kyoto encyclopedia of genes and genomes
KO: KEGG orthology
Lus: *Linum usitatissimum*
Mir: microRNA
MiRNA: microRNA
NCBI: National Center for Biotechnology Information
NSS: neutral salt stress
qRT-PCR: quantitative RT-PCR
SRA: short read archive
sRNA: small RNA
TFs: transcription factors
U: uridine
UV: ultraviolet
