# Supplementary figures and images for: Identification and characterization of miRNAs and targets in flax (Linum usitatissimum) under saline, alkaline, and saline-alkaline stresses
Source: BMC Plant Biol. 2016 May 27;16:124. doi: 10.1186/s12870-016-0808-2 (PMC4884397; doi:10.1186/s12870-016-0808-2)

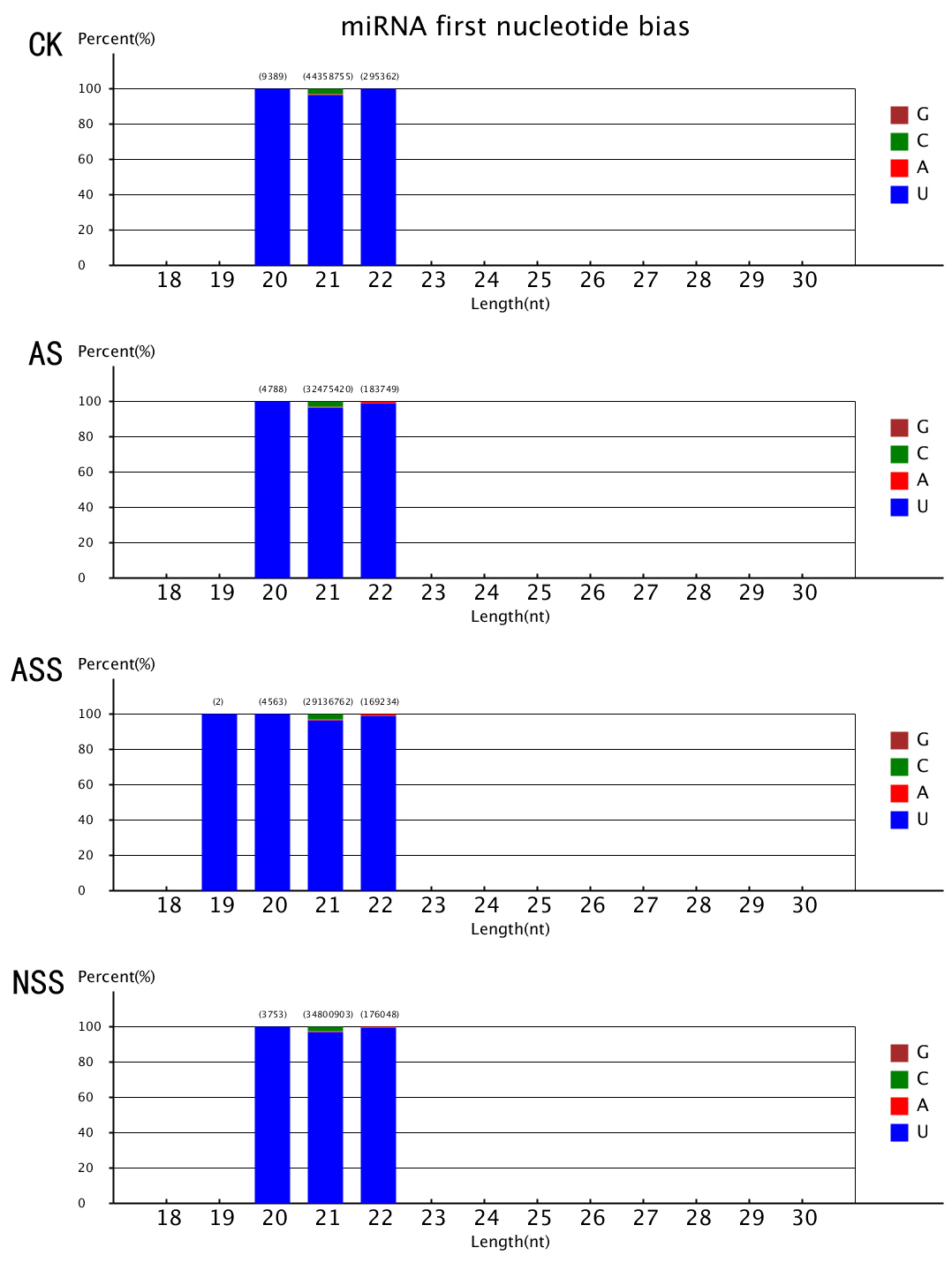

Supplement: Additional file 1: — First nucleotide bias of 18–30 nt sRNA tags. (TIF 4533 kb) [file 12870_2016_808_MOESM1_ESM.tif]
