# Supplementary material for: Identification and characterization of miRNAs and targets in flax (Linum usitatissimum) under saline, alkaline, and saline-alkaline stresses
Source: BMC Plant Biol. 2016 May 27;16:124. doi: 10.1186/s12870-016-0808-2 (PMC4884397; doi:10.1186/s12870-016-0808-2)
Supplement: Additional file 7: — GO ontology statistics of target genes of differential expressed known miRNAs and novel miRNAs. (PDF 275 kb) [file 12870_2016_808_MOESM7_ESM.pdf]

A

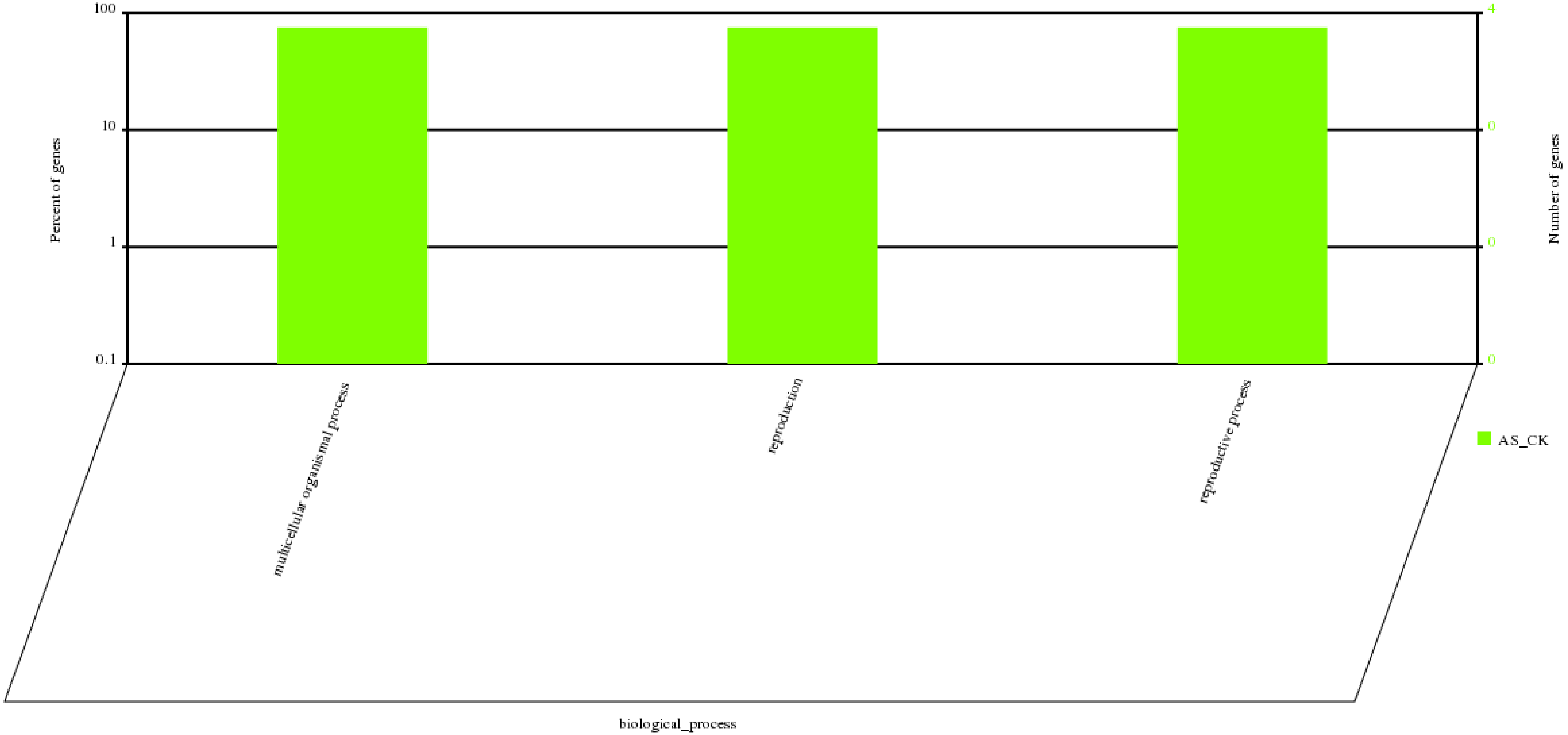

100
10
1
0.1
Percent of genes
4
0
0
0
Number of genes
multicellular organismal process
reproduction
reproductive process
AS_CK
biological_process

B

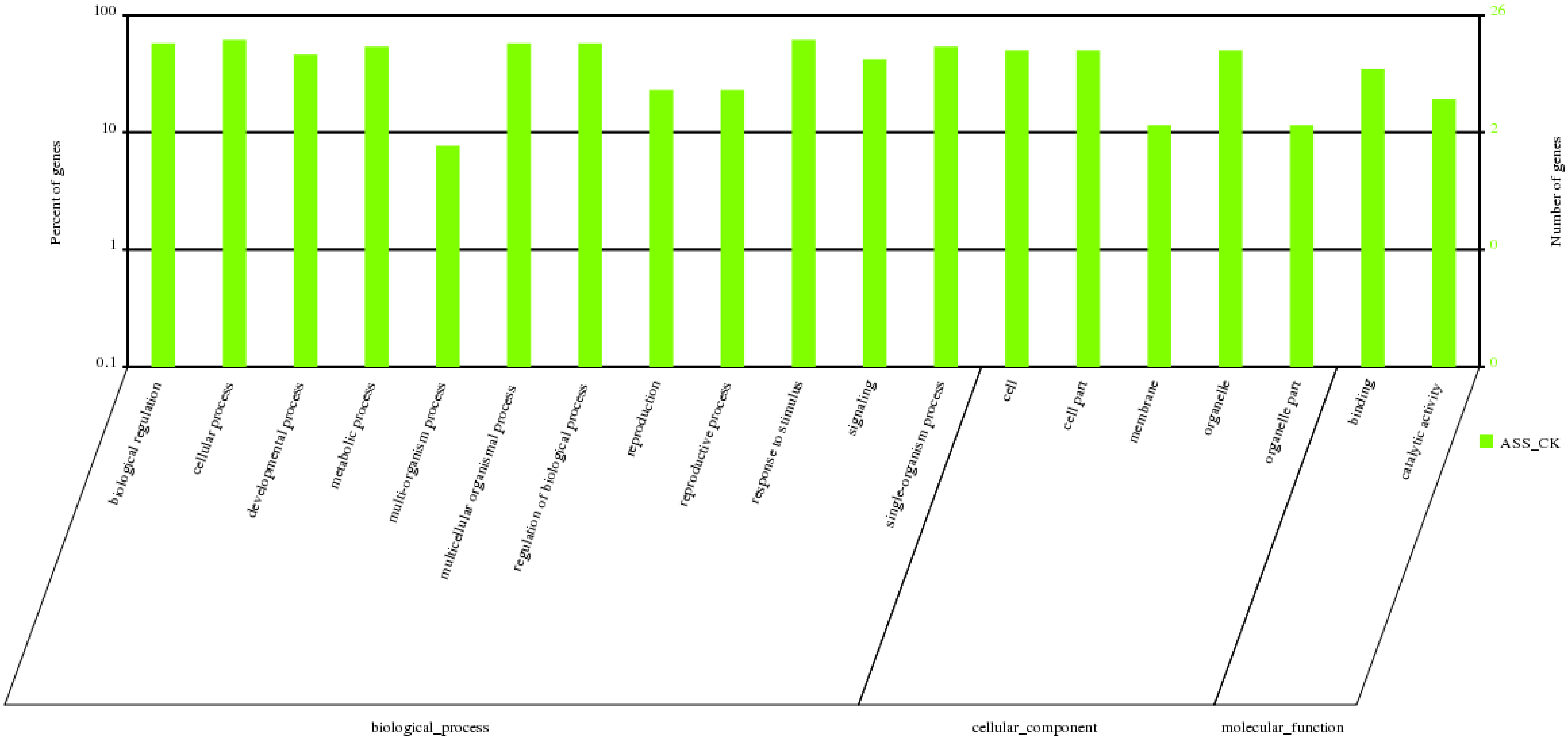

100
10
1
0.1
Percent of genes
26
2
0
0
Number of genes
biological regulation
cellular process
developmental process
metabolic process
multi-organism process
multicellular organismal process
regulation of biological process
reproduction
reproductive process
response to stimulus
signaling
single-organism process
cell
cell part
membrane
organelle
organelle part
binding
catalytic activity
ASS_CK
biological_process
cellular_component
molecular_function

C

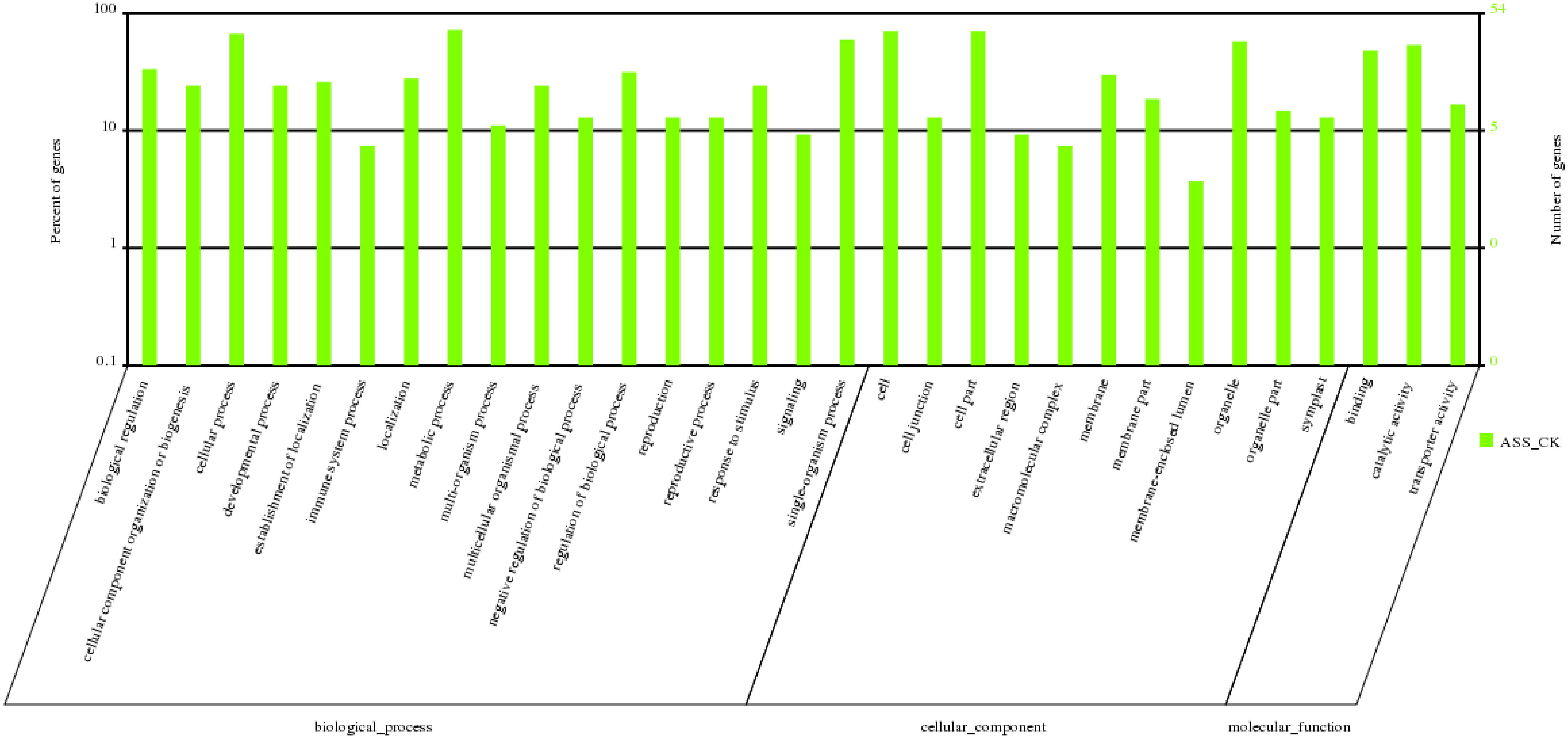
100
10
1
0.1
Percent of genes
54
5
0
0
Number of genes
biological regulation
cellular component organization or biogenesis
cellular process
developmental process
establishment of localization
immune system process
localization
metabolic process
multi-organism process
multicellular organismal process
negative regulation of biological process
regulation of biological process
reproduction
reproductive process
response to stimulus
signaling
single-organism process
cell
cell junction
cell part
extracellular region
macromolecular complex
membrane
membrane part
membrane-enclosed lumen
organelle
organelle part
symplast
binding
catalytic activity
transporter activity
ASS_CK
biological_process
cellular_component
molecular_function

D

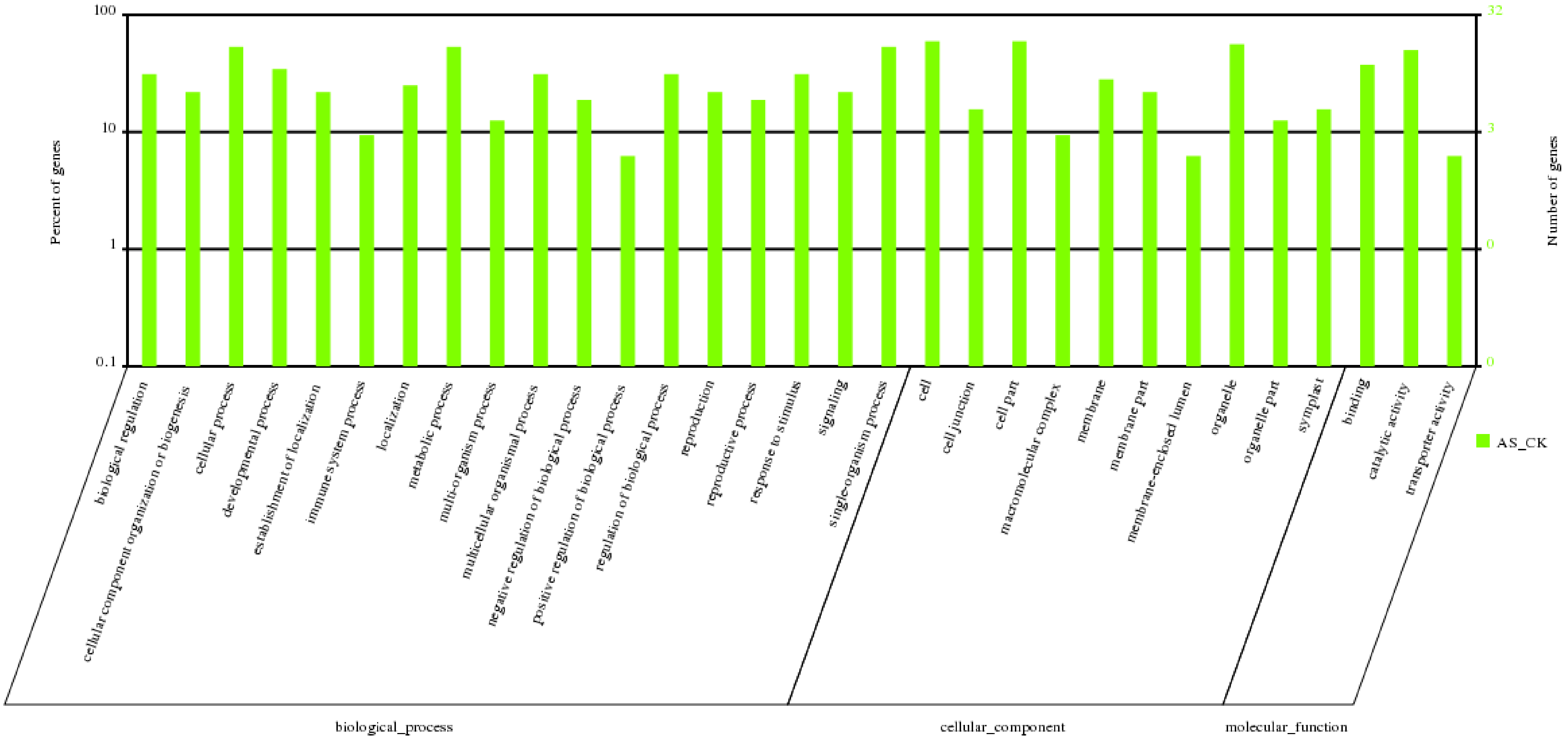

100
10
1
0.1
Percent of genes
32
3
0
0
Number of genes
biological regulation
cellular component organization or biogenesis
cellular process
developmental process
establishment of localization
immune system process
localization
metabolic process
multi-organism process
multicellular organismal process
negative regulation of biological process
positive regulation of biological process
regulation of biological process
reproduction
reproductive process
response to stimulus
signaling
single-organism process
cell
cell junction
cell part
macromolecular complex
membrane
membrane part
membrane-enclosed lumen
organelle
organelle part
symplast
binding
catalytic activity
transporter activity
AS_CK
biological_process
cellular_component
molecular_function

E

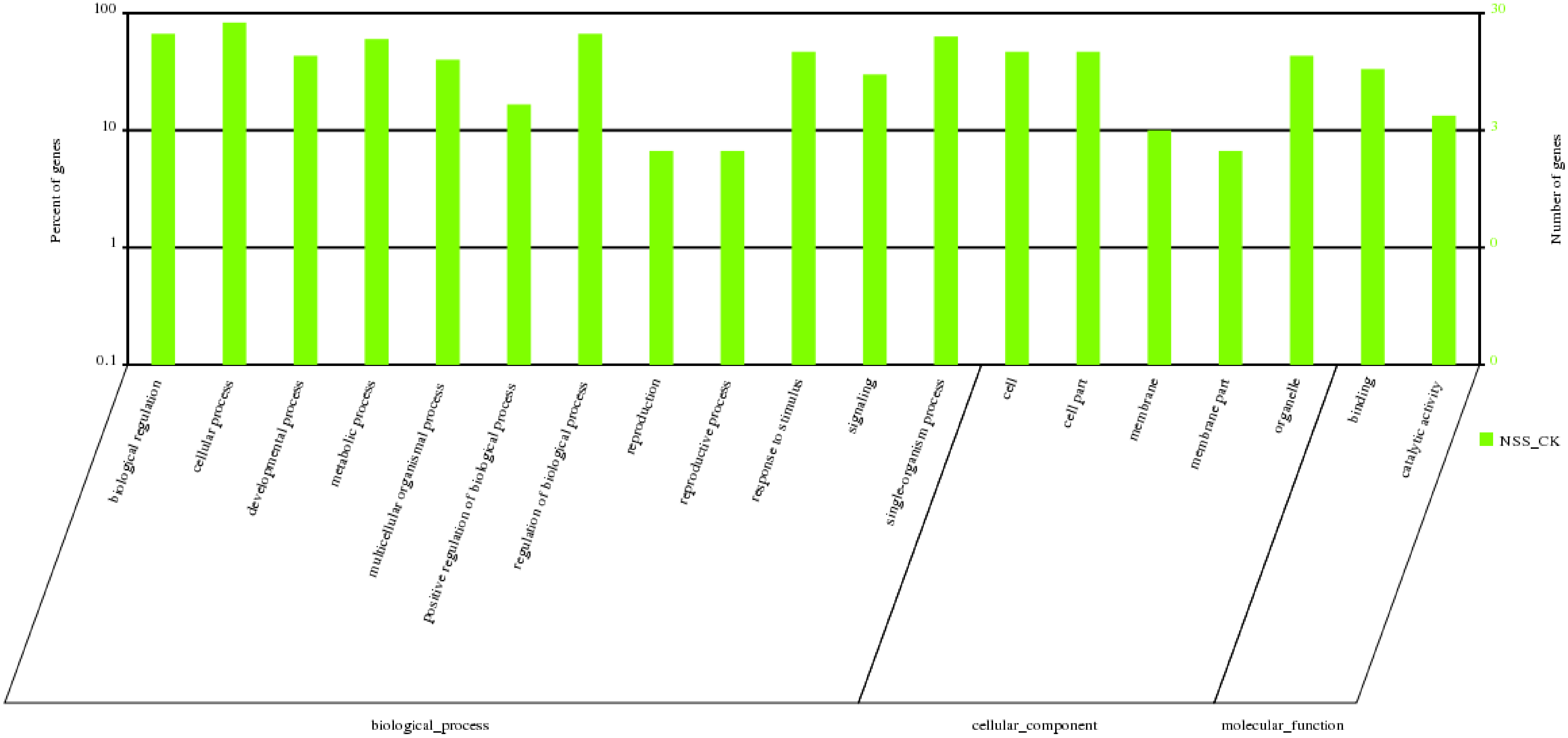

Percent of genes
100
10
1
0.1
Number of genes
30
3
0
0
biological regulation
cellular process
developmental process
metabolic process
multicellular organismal process
positive regulation of biological process
regulation of biological process
reproduction
reproductive process
response to stimulus
signaling
single-organism process
cell
cell part
membrane
membrane part
organelle
binding
catalytic activity
NSS_CK
biological_process
cellular_component
molecular_function

F

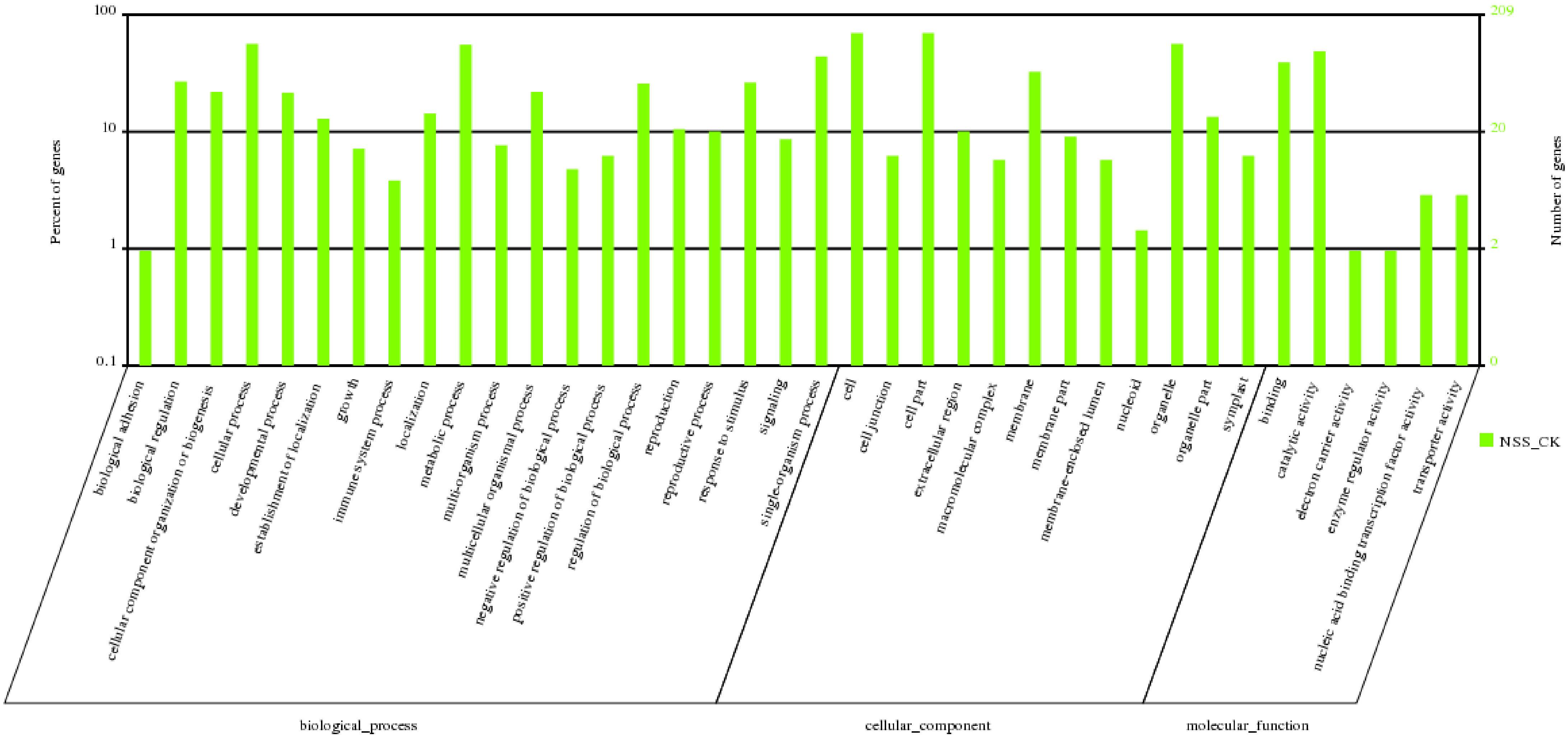

Additional file 7 GO ontology statistics of target genes of differential expressed known miRNAs and novel miRNAs

In this ontology, 'cellular location,' 'biological process' and 'molecular function' are treated as independent attributes. A-C. Known miRNA targets in AS_CK, AS2_CK, NSS_CK, respectively; D-F. Novel miRNA targets in AS_CK, AS2_CK, NSS_CK, respectively.
